# Translation and Validation of the Rhinitis Control Assessment Test (RCAT) for Allergic Rhinitis in Urdu Language

**DOI:** 10.1055/s-0046-1818632

**Published:** 2026-05-05

**Authors:** Maira Adeel, Ahmad Nawaz, Hamza Ahmed Larik, Maheen Pyarali, Ammad Ali, Ramsha Tariq, Najeebullah Arbani, Haissan Iftikhar

**Affiliations:** 1 Department of Ear, Nose, and Throat—Head and Neck Surgery, Liaquat National Hospital and Medical College, Karachi, Pakistan; 2 Department of Surgery, Liaquat National Hospital and Medical College, Karachi, Sindh, Pakistan.; 3 Department of Surgery, Aga Khan University Hospital, Karachi, Sindh, Pakistan.

**Keywords:** RCAT, Urdu RCAT, allergic rhinitis, translation, validation

## Abstract

**Introduction:**

Allergic rhinitis (AR) is an IgE-mediated inflammatory reaction of the nasal mucosa triggered by various allergens, leading to nasal congestion, rhinorrhoea, sneezing, nasal itching, ocular redness, lacrimation, and postnasal dripping. This condition can also cause sleep disturbances and fatigue, which can affect productivity at work and academic performance, therefore posing substantial economic burden. Positive outcomes rely on efficacy of medications, potential adverse effects of medication, treatment response, and overall prognosis.

**Objective:**

To investigate the utility of the Rhinitis Control Assessment Test (RCAT) as a clinical tool in Urdu for evaluating and monitoring allergic rhinitis.

**Methods:**

The RCAT is a self-administered questionnaire consisting of six questions addressing specific rhinitis symptoms.

**Results:**

The study included 60 (35 male and 25 female) patients with ages ranging from 18 to 55 (mean: 35) years. The RCAT demonstrated acceptable internal consistency with a Cronbach's alpha of 0.759. Interitem correlations supported that the questionnaire items reflected related constructs. Paired sample t-tests showed statistically significant improvements in all RCAT items posttreatment ( *p* < 0.001), except for the compliance-related item (q5; *p* = 0.166). The overall score increased significantly (mean difference = 9.28; *p* < 0.001), indicating improved rhinitis control.

**Conclusion:**

The RCAT has proven to be a valuable tool for assessing allergic rhinitis control. Its ease of use and adaptability across languages make it an excellent choice for routine monitoring and management of AR. The successful application of RCAT in Urdu further enhances its accessibility and effectiveness for native speakers.

## Introduction

Allergic rhinitis has a high prevalence, affecting ¼ of the world's population. According to Asim et al., 1 the data from patients registered in allergic infection center Islamabad from 2012 to 2014 consisted of a mix of cases of allergic rhinitis and asthma (44.86%), whereas allergic rhinitis alone was 34.1% in the province of Punjab. Reported cases in Khyber Pakhtunkhwa (KPK) totaled 26.29% . Allergic rhinitis was most common in Sindh, with a prevalence of 44.92%. In Baluchistan, urticaria was the most prevalent atopic disease, while bronchial asthma, allergic rhinitis, and mixed cases of allergic rhinitis and asthma had the respective rates of 34.23, 2.28.67, and 19.12%. 1

There is no recent comprehensive database of allergic rhinitis prevalence in Pakistan. However, several small-scale studies conducted in hospitals and schools have reported on the condition. One of the studies conducted in a Karachi-based school found a high incidence of allergies, with allergic rhinitis affecting 28.5% of school children. 2 Another study conducted among healthcare workers in a tertiary care hospital concluded that allergic rhinitis was present in 19.2% of healthcare workers, with female workers being 2.2 times more prone to this condition. 3

Allergic rhinitis (AR) is a chronic, noncontagious disease that significantly impacts quality of life. 4 5 It's an IgE mediated inflammatory reaction of the nasal mucosa triggered by various allergens, leading to nasal congestion, rhinorrhea, sneezing, nasal itching, ocular redness, lacrimation, and postnasal dripping. Additionally, it causes sleep disturbances and fatigue, affecting productivity at work and academic performance, thereby posing a substantial economic burden. 6 7 8 Disease control depends on medication efficacy, potential adverse effects, treatment response, exacerbation impact, and overall prognosis. 9

The Rhinitis Control Assessment Test (RCAT) is an accessible and efficient tool for evaluating rhinitis control at home. It generates a score that allows patients to objectively assess their condition, empowering them to seek better treatment and improve disease management. For physicians, RCAT provides a prompt assessment in busy clinical settings, serving as an excellent tool for screening allergic rhinitis and monitoring the condition over time. The recorded scores can be compared with previous results aiding therapeutic decisions. 9 10

Originally validated in American English, RCAT has since been translated and validated in multiple languages worldwide, including Spanish, Portuguese, Bulgarian, and Thai. 11 12 13 14 It is a reliable, simple, and brief self-administered questionnaire suitable for both short- and long-term monitoring of allergic rhinitis. 10 The test functions as a follow-up tool for patient-reported outcomes, comprising six questions addressing specific symptoms. 15 16 17 18 Matricardi et al. 11 with their translation of the RCAT questionnaire into Portuguese, demonstrated that it was well understood by allergic rhinitis patients and had strong discriminatory power. 11 19 20 21

The objective of our study was to translate, validate and adapt the RCAT questionnaire for use by Urdu-speaking patients throughout South Asia, a population of approximately 230 million. 22

## Methods

### Study Design and Setting

A prospective cross-sectional study was conducted for a period of 6 months for patients presenting with allergic rhinitis to our Ear, Nose, and Throat (ENT) clinic in a tertiary care center. The study was approved by the Ethical Review Board.

### Inclusion Criteria

All the patients who presented to the ENT clinic with nasal obstruction, nasal discharge, and sneezing due to an external allergen or factor were included.

### Exclusion Criteria

Patients who did not consent to complete the questionnaire, and those who were unable to read and understand Urdu language were excluded from the study.

### Tool Development Procedure

**Fig. 1:**
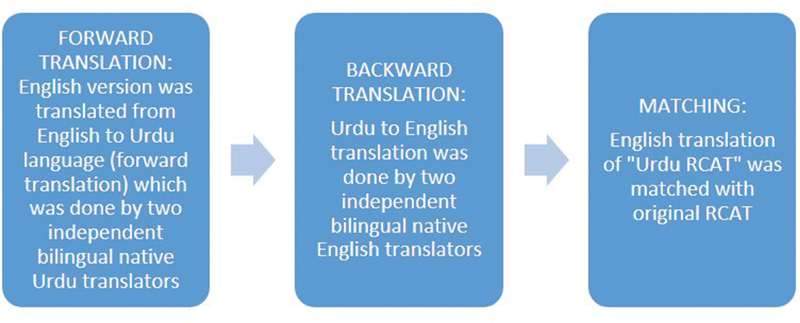
Tool development process.

The RCAT English version ( Table 1 ) was translated into Urdu (forward translation) which was done by two independent bilingual native Urdu translators. Then backward translation of the “RCAT Urdu translation” to English was done by two independent bilingual native English translators and was compared to the original RCAT English version. An overview of the translation process is illustrated in Fig. 1 .

**Table 1:** English version of the Rhinitis Control Assessment Test (RCAT)

1. During the past week, how often did you have nasal congestion?				
Never	Rarely	Sometimes	Often	Extremely often
☐ 5	☐ 4	☐ 3	☐ 2	☐ 1
2. During the past week, how often did you sneeze?				
Never	Rarely	Sometimes	Often	Extremely often
☐ 5	☐ 4	☐ 3	☐ 2	☐ 1
3. During the past week, how often did you have watery eyes?				
Never	Rarely	Sometimes	Often	Extremely often
☐ 5	☐ 4	☐ 3	☐ 2	☐ 1
4. During the past week, to what extent did your nasal or other allergy symptoms interfere with your sleep?				
Never	Rarely	Sometimes	Often	Extremely often
☐ 5	☐ 4	☐ 3	☐ 2	☐ 1
5. During the past week, how often did you avoid any activities (for example visiting a house with a dog or cat, gardening) because of your nasal or other allergy symptoms?				
Never	Rarely	Sometimes	Often	Extremely often
☐ 5	☐ 4	☐ 3	☐ 2	☐ 1
6. During the past week, how well were your nasal or other allergy symptoms controlled?				
Completely	Very	Somewhat	Poorly	Not at all
☐ 5	☐ 4	☐ 3	☐ 2	☐ 1

### Data Collection Procedure

Patients visiting the outpatient department were diagnosed for allergic rhinitis by a combination of a detailed medical and family history, physical exam, potential triggers, and performing allergy tests. Informed consent was taken from the patients after explaining to them all the research's prerequisites. Enrolled patients were asked to physically fill out the RCAT Urdu version ( Table 2 ). Demographic data such as name, age, and medical record number were all recorded in English in a separate Performa (Pretreatment). These patients were then prescribed conservative treatment which included a steroid nasal spray (mometasone furoate, 2 puffs/day for weeks), and a systemic anti-allergic (desloratadine, one tablet/day) for a period of four weeks. They were instructed to follow up after 4 weeks and the RCAT questionnaire was completed post treatment.

**Table 2:** Urdu version of the Rhinitis Control Assessment Test (RCAT)

پچھلے ایک ہفتے میں آپ نے کتنی بار ناک بند محسوس کیا؟ ۱.				
کبھی نہیں	کبھی کبھار	بعض اوقات	اکثر	زیادہ تر
☐5	☐4	☐3	☐2	☐1
پچھلے ایک ہفتے میں آپ کو کتنی بار چھینکیں آئیں؟ ۲.				
کبھی نہیں	کبھی کبھار	بعض اوقات	اکثر	زیادہ تر
☐5	☐4	☐3	☐2	☐1
پچھلے ایک ہفتے میں آپ کو کتنی مرتبہ انکھوں سے پانی آنے کی شکایت ہوئی؟ ۳.				
کبھی نہیں	کبھی کبھار	بعض اوقات	اکثر	زیادہ تر
☐5	☐4	☐3	☐2	☐1
پچھلے ایک ہفتے میں اپکی ناک اور الرجی کی دیگر علامت کے باعث نیند کتنی متاثر ہوئی؟ ۴.				
کبھی نہیں	کبھی کبھار	بعض اوقات	اکثر	زیادہ تر
☐5	☐4	☐3	☐2	☐1
چھلے ایک ہفتے میں کتنی مرتبہ آپ نے ایسے کاموں جیسے جانوروں کے قریب جانا یا باغانی سے پرہیز کیا؟ ۵.				
کبھی نہیں	کبھی کبھار	بعض اوقات	اکثر	زیادہ تر
☐5	☐4	☐3	☐2	☐1
پچھلے ایک ہفتے میں اپکی ناک اور الرجی کی دیگر علامت میں بہتری آئی؟ ۶.				
زیادہ تر	اکثر	بعض اوقات	کبھی کبھار	نہیں کبھی
☐5	☐4	☐3	☐2	☐1

## Data Analysis

Data was recorded and analyzed using IBM SPSS Statistics (IBM Corp.) version 30. Measures such as mean, minimum, and maximum values for continuous variables like age, as well as frequencies and percentages for categorical variables, such as gender, were recorded.

Cronbach's alpha was employed to calculate the reliability of RCAT Urdu version, with values over 0.700 being considered significant.

Interitem correlation analysis was conducted to examine the degree to which individual items on the RCAT were related to one another. This analysis helps determine whether all questions measure the same underlying construct which in this case is rhinitis control. Positive Pearson correlation coefficients indicate correlation between items of questionnaire.

To assess the effectiveness of treatment and measure changes in symptom scores, a paired sample *t* -test was used. This test compares the means of two related groups: pre- and posttreatment scores, to determine whether there is a statistically significant difference in responses after intervention. The paired *t* -test was chosen specifically because it accounts for the dependency between repeated measures on the same subjects. A *p* -value of less than 0.05 was considered statistically significant.

## Results

**Fig. 2:**
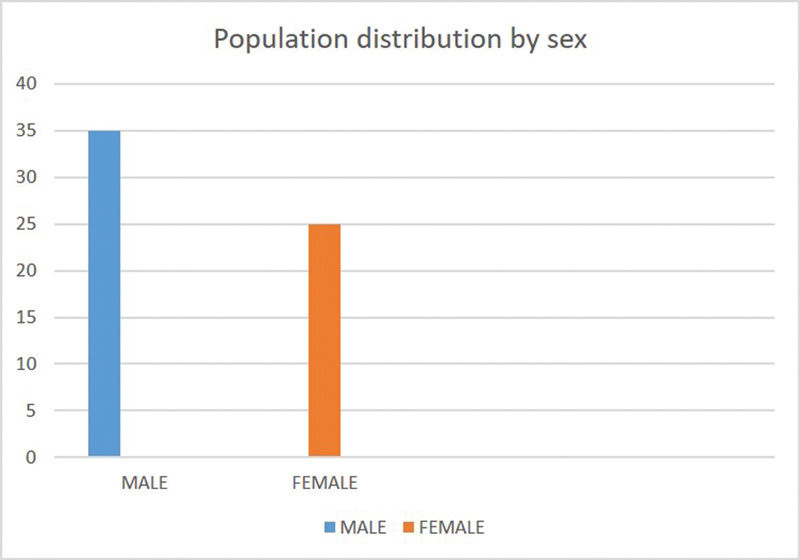
Urdu version of the Rhinitis Control Assessment Test (RCAT).

The study included 60 (35 male and 25 female) patients with ages ranging from 18 to 55 (mean: 35) years ( Fig. 2 ).

A Cronbach's Alpha value of 0.759 was obtained ( Table 3 ), which exceeds the commonly accepted threshold of 0.700. The same value was obtained for standardized items showing reliability even when item variances are accounted for. The analysis was based on six items in the RCAT, each representing a distinct but related symptom of allergic rhinitis and these results show that these items function well together.

**Table 3:** Reliability statistics

Cronbach's Alpha	Cronbach's Alpha based on standardized items	Items (N)
0.759	0.759	6

Interitem correlation was measured to assess if all the questions and their relationship with each other were reflecting the same construct. In the Pretreatment scores, most interitem correlations ranged from moderate to strong (e.g., q1 and q4: *r* = 0.677; q2 and q3: *r* = 0.472), indicating that the items are generally measuring related aspects of allergic rhinitis control. However, some correlations were relatively low (e.g., q2 and q6: *r* = 0.061; q1 and q6: *r* = 0.148), suggesting that question 6 may be measuring a different or less closely related symptom ( Table 4 ). In the posttreatment scores, the overall correlation levels were lower compared to pretreatment, though still within an acceptable range. The strongest correlation was between questions 1 and 2 ( *r* = 0.409), while some item pairs showed very low or even negative correlations (e.g., q4 and 6: *r* = -0.032), as presented in ( Table 5 ), which may reflect changes in symptom dynamics after treatment or variability in patient perception. Overall, the pattern of correlations supports that the RCAT items are reasonably interrelated and appropriate for assessing the same underlying construct, such as rhinitis control, but it also suggests that question 6 may need further review due to consistently weak correlation with other items.

**Table 4:** Interitem correlation matrix (pretreatment)

	q1	q2	q3	q4	q5	q6
q1	1.000	0.350	0.317	0.677	0.379	0.148
q2	0.350	1.000	0.472	0.331	0.408	0.061
q3	0.317	0.472	1.000	0.430	0.329	0.283
q4	0.677	0.331	0.430	1.000	0.440	0.220
q5	0.379	0.408	0.329	0.440	1.000	0.324
q6	0.148	0.061	0.283	0.220	0.324	1.000

**Table 5:** Interitem correlation matrix (posttreatment)

	q1	q2	q3	q4	q5	q6
q1	1.000	0.409	0.391	0.262	0.115	0.235
q2	0.409	1.000	0.243	0.085	0.078	0.101
q3	0.391	0.243	1.000	0.356	0.103	0.185
q4	0.262	0.085	0.456	1.000	0.320	0.032
q5	0.115	0.078	0.103	0.320	1.000	0.126
q6	0.235	0.101	0.185	0.032	0.126	1.000

A paired sample t-test was conducted to compare the pre- and posttreatment scores of the RCAT questionnaire. The analysis revealed a statistically significant difference ( *p* < 0.001) between the pre- and postintervention scores for all items, indicating overall improvement in patient-reported symptoms following treatment. However, question 5, which assessed patient compliance, did not demonstrate a statistically significant change ( *p* = 0.166). This item reflects individual behavioral tendencies and compliance ( Table 6 ).

**Table 6:** Paired samples *t* -test

	Paired differences (mean)	SD	SEM	*t*	df	*p* -value
q1 post– pre	2.26	1.23	0.159	14.2	59	0.000
q2 post–pre	2.483	1.033	0.133	18.618	59	0.000
q3 post–pre	1.350	1.412	0.182	7.405	59	0.000
q4 post–pre	1.050	1.534	0.198	5.302	59	0.000
q5 post–pre	0.267	1.471	0.190	1.404	59	0.166
q6 post–pre	1.867	1.467	0.189	-9.858	59	0.000
post–prescore	9.283	3.971	0.513	18.110	59	0.000

**Abbreviations:** df, degrees of freedom; post, posttreatment; pre, pretreatment; SD, standard deviation; SEM, standard error of the mean.

## Discussion

In the management of chronic diseases, various tools have been used. These tools serve various purposes, including patient screening in primary care settings and aiding with specialist medical management.

Despite breakthroughs in our understanding of the pathogenesis of allergic rhinitis and the accessibility of efficient treatment, many patients continue to find it difficult to control their condition. 1 2 For the purpose of evaluating whether care is optimized and modifying treatment plans to reach the objective of well-controlled rhinitis, precise evaluation of allergic rhinitis control is vital. As a result, the RCAT was created as a brief, patient-rated instrument to assist in identifying individuals who require additional evaluation and better management. Notably, there are further patient-rated screening tools that have been validated and translated into numerous languages, all of which assess the degree of disease control for prevalent chronic disorders such as allergic rhino conjunctivitis 3 and chronic rhinosinusitis/nasal polyposis (22-Item Sinonasal Outcome Test, SNOT-22). 4

There were five categories of questions selected: symptoms, interference in activities, limitations, rhinitis control, and medication use. The initial version of the questionnaire underwent evaluation following application to an extensive number of patients, and the most pertinent questions were found using logistic regression analysis. The final form contained six items. 5

The RCAT is designed for patients ≥ 18-years-old with allergic and nonallergic rhinitis. Each of the six items is rated on a five-point scale with a possible score from 6 to 30. Question no.5 in the questionnaire is more related to a patient's own precautionary measures habits and routine. It doesn't affect the score much on the basis of treatment regime but was still included because of the element of compliance.

Statistical data identified a cut-off point of 21 as a border between controlled and uncontrolled allergic and nonallergic rhinitis. As such, a score of 21 or less is used to identify allergic versus nonallergic rhinitis.

The obligatory part of the linguistic validation procedure is cognitive debriefing. To make it obvious whether the suggested version is correctly understood, it is tested among a group of patients. It is thought to be a challenge, as validation is not as simple as translation. In fact, it is about addressing expressions from one language to another for a specific variety of age, culture, and psychological characteristics.

Translations in our research were performed by four authorized translators (registered), two translating the questionnaire to Urdu and the others retranslating it back to English, confirming that the Urdu version of RCAT is correct.

Next, the questionnaire was tested among a group of patients to consider the linguistic validation. Before beginning, the patients were counseled on the purpose of this study.

## Conclusion

In the present publication, we demonstrate a validated questionnaire for rhinitis control assessment in Urdu. It makes it possible to assess the control of allergic rhinitis in Pakistani patients. Its application can complement the control evaluation by physicians of patients with rhinitis.
